# Pneumomediastinum caused by occult paraquat poisoning: Case report

**DOI:** 10.1097/MD.0000000000013745

**Published:** 2018-12-21

**Authors:** Peng Deng, Yao Chen, Hong Li, Zhi Wan

**Affiliations:** Emergency Department of West China Hospital Sichuan University, Chengdu, China.

**Keywords:** hemoperfusion, herbicide, paraquat, pneumomediastinum, severity index of paraquat poisoning, toxication

## Abstract

**Rationale:**

Paraquat is a widely applied contact herbicide that is highly poisonous. About 20% of patients with paraquat poisoning develop pneumomediastinum as a complication with a mortality rate of almost 100%.

**Patient concerns:**

A 15-year-old boy presented with a 1-month history of retrosternal chest pain with no obvious cause. High-resolution computed tomography showed pneumomediastinum.

**Diagnoses:**

After all likely causes of pneumomediastinum were eliminated, the diagnosis of occult paraquat poisoning was made when serum paraquat concentration was revealed at 467.40 ng/mL, despite the patient's denial of ingestion or contact.

**Interventions:**

Hemoperfusion, intravenous glucocorticoid, and ulinastatin was administered for 3 days with other routine treatment against paraquat poisoning. The serum paraquat concentration decreased to zero.

**Outcomes:**

Despite the general high mortality and poor prognosis of paraquat poisoning, the patient recovered and was completely asymptomatic at his 3-month follow-up.

**Lessons:**

Paraquat poisoning should be suspected as a differential diagnosis when patients present with pneumomediastinum without recognizable cause.

## 1 Introduction

Paraquat (1,1′-dimethyl-4,4′-bipyridinium dichloride) is a widely applied contact herbicide that is highly poisonous. It was introduced in the 1960s and is used in over 130 countries today. When ingested or inhaled, it can cause fatal poisoning, with a mortality rate as high as 60% to 80%.^[1,2]^ There is no effective treatment or antidote against paraquat poisoning.

Pneumomediastinum is the abnormal presence of air or other gas in the mediastinum. It is most commonly caused by chest trauma, rupture of the esophagus, or pulmonary diseases that lead to alveolar rupture. Previous study has shown that about 20% of patients with paraquat poisoning develop pneumomediastinum as a complication. The mortality rate with this complication is almost 100%.^[3,4]^

## 2 Case description

A 15-year-old boy sought care at the emergency department with a 1-month history of retrosternal chest pain with no obvious cause. The chest pain was accompanied by shortness of breath and was exaggerated during inspiration. The patient had no fever, cough, dysphagia, choking, dizziness, anorexia, fatigue, abdominal pain, or diarrhea. Physical examination was almost negative except for subcutaneous emphysema over the neck region.

**Figure 1:**
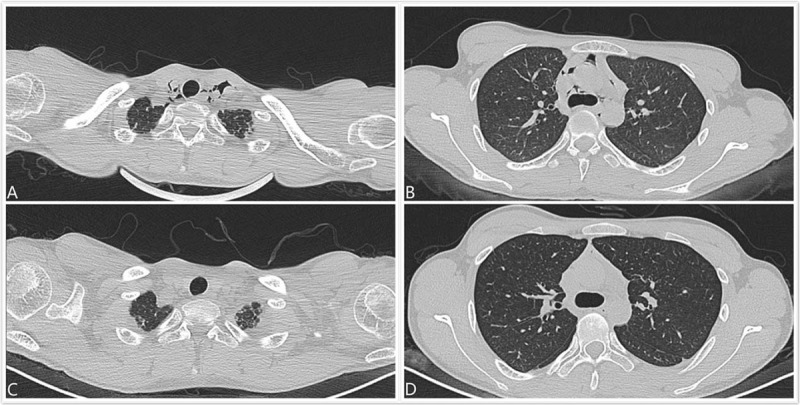
HRCT of the lung upon admission, showing gas in the (A) thoracocervical area and (B) mediastinum, and (C, D) significantly decreased gas in the same area after treatment at day 8 of admission.

**Figure 2:**
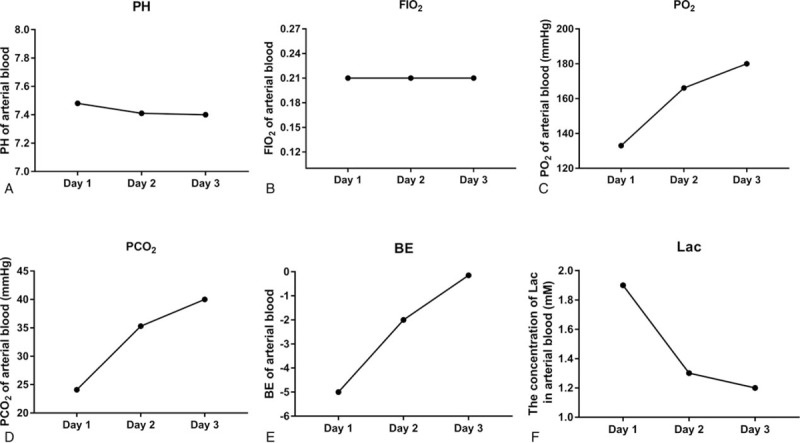
Arterial blood gas at day 1, 2, and 3 of admission, including (A) pH, (B) FIO_2_, (C) pO_2_, (D) pCO_2_, (E) base excess (BE), and (F) lactate (Lac).

Thoracic high-resolution computed tomography (HRCT) of the patient (Fig. 1A, B) showed gas accumulation in the thoracocervical region and mediastinum. No abnormality was found in the trachea, bronchi, or lung parenchyma. Bedside arterial blood gas analysis showed mild metabolic acidosis with normal oxygen saturation at 99.6% on normal air (Fig. 2). Routine blood analysis did not reveal any abnormality in complete blood count, liver and kidney function, coagulation status, cardiac enzymes, tumor markers (alpha-fetoprotein, CEA, CA125, Cyfra 21-1), lactate, or procalcitonin. Hepatitis, syphilis, and human immunodeficiency virus (HIV) screening tests were all negative. Esophagus iodine oil radiography, gastroscopy, and fiber bronchoscopy were performed to help identify the source of air in the mediastinum, but nothing positive was found. Repeated medical histories failed to reveal any cause of the patient's pneumomediastinum.

All likely causes of pneumomediastinum were eliminated. On the third day of admission, the serum paraquat concentration was measured, despite the patient's denial of ingestion or contact. The serum paraquat concentration was found to be 467.40 ng/mL, and thus, occult paraquat poisoning was diagnosed. Standard treatment against paraquat poisoning was immediately initiated with the application of hemoperfusion, intravenous glucocorticoid (methylprednisolone 80 mg, 2×/day), and ulinastatin (100,000 IU, 3×/day).

**Figure 3:**
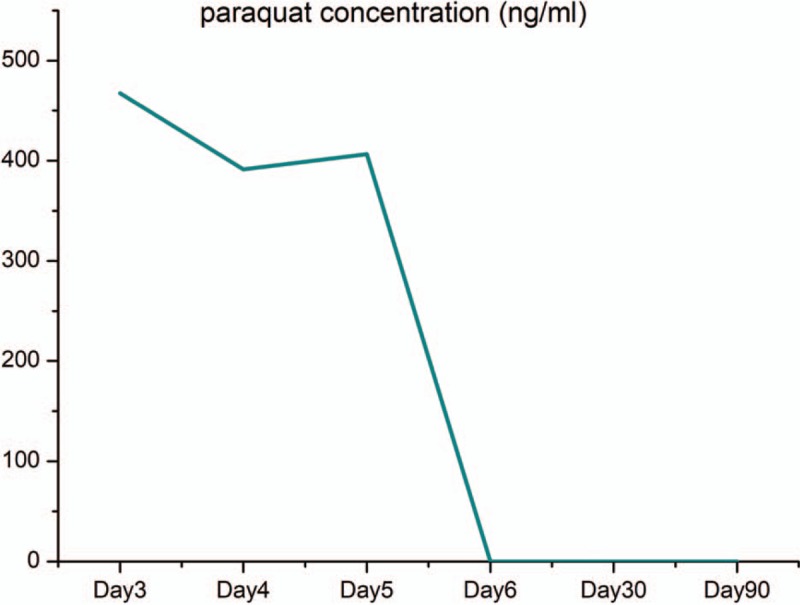
Blood paraquat concentration at diagnosis at day 3 of admission and after treatment.

The serum paraquat concentration was carefully monitored daily and fell to nil on the sixth day after admission (Fig. 3). The symptoms of chest pain and shortness of breath were significantly relieved. Thoracic HRCT on the eighth day after admission (Fig. 1C, D) showed that gas accumulation in the thoracocervical region and mediastinum was significantly absorbed. On the tenth day, the patient was discharged from the hospital.

When followed up at 1 and 3 months after discharge, the patient experienced no more chest pain, and HRCT of the lungs was normal. His serum paraquat concentration remained nil and liver and kidney functions were stable and normal.

## 3 Discussion

This is a very rare case of subacute occult paraquat poisoning, characterized by the single chief complaint of chest pain and the unique clinical presentation of pneumomediastinum. Pneumomediastinum and subcutaneous emphysema have been recognized as serious complications of paraquat poisoning.^[3]^ Yet, to our best knowledge, this case of subacute occult paraquat poisoning without any history of paraquat exposure is one of a kind. No similar case has been reported in the published literature.

In this case, paraquat poisoning was difficult to recognize because there was no clue in the history, no erosion in the patient's mouth or pharynx, and no hepatic or renal dysfunction, which is common in paraquat poisoning. Pneumomediastinum with subcutaneous emphysema was the only presentation. The pneumomediastinum may have been due to gas leakage subsequent to injury of the air passage, as no abnormality was seen on esophagus iodine oil radiography and gastroscopy. There was no foreign body or other lesion on bronchoscopy.

The patient's symptoms of chest pain for 1 month before hospital admission led to a severity index of paraquat poisoning (SIPP = plasma paraquat concentration at admission, mg/L × time from poisoning to admission, hours) of 331.2 mg h/L. This is much higher than the 50 mg h/L that has been associated with poor prognosis and high mortality.^[5]^

Pneumomediastinum and subcutaneous emphysema after paraquat poisoning may be due to esophageal erosion and perforation from the corrosive effect of paraquat.^[6]^ Another proposed cause is air leakage from ruptured alveoli along the peribronchovascular interstitium, due to paraquat-induced alveolar exudation, fibrosis, and increased alveolar tension and shear forces.^[7]^ Additional contributing mechanisms include esophageal damage due to severe vomiting after poisoning or repeated gastric lavage, damage to the air passage from mechanical ventilation, or other factors.

As far as we know, no antidote for paraquat is available. Some studies have suggested that hemoperfusion may be an appropriate treatment for paraquat poisoning, and that glucocorticoid can be effective.^[8]^ Moreover, it has been reported that antioxidants can prevent the progress of pulmonary fibrosis.^[9]^ For our patient, all of these treatment methods were applied with a good outcome.

This case report is limited by the lack of discovery of the source of paraquat poisoning, and no tracheal or bronchial erosion was found on the bronchoscopy to reveal the pathology of pneumomediastinum.

## 4 Conclusion

When pneumomediastinum is present without any recognizable cause, paraquat poisoning should be considered. The diagnosis of paraquat poisoning is confirmed based on serum paraquat concentration. Comprehensive therapy should be started immediately, as full recovery is possible, despite the general poor prognosis of the condition.

## 5 Patient consent statement

Written informed consent was obtained from the patient's guardians for publication of the case.

## Acknowledgments

The authors extend their sincere gratitude to all colleagues at the emergency department. This work was supported financially by grants from Science Foundation of Science and Technology Department in Sichuan (No. 2017SZ0190) and The National Key Research and Development Program of China (No. 2017YFC0908702).

## Author contributions

**Conceptualization:** Peng Deng, Yao Chen, Hong Li, Zhi Wan.

**Data curation:** Peng Deng, Yao Chen, Hong Li, Zhi Wan.

**Project administration:** Peng Deng, Yao Chen, Hong Li, Zhi Wan.

**Resources:** Peng Deng.

**Supervision:** Peng Deng, Yao Chen, Hong Li, Zhi Wan.

**Validation:** Peng Deng, Yao Chen, Hong Li, Zhi Wan.

**Visualization:** Peng Deng, Yao Chen, Hong Li, Zhi Wan.

**Writing – original draft:** Peng Deng, Yao Chen, Hong Li, Zhi Wan.

**Writing – review & editing:** Peng Deng, Yao Chen, Hong Li, Zhi Wan.
